# Parasitemia and antibody response to benznidazole treatment in a cohort of patients with chronic Chagas disease

**DOI:** 10.3389/fpara.2023.1235925

**Published:** 2023-09-05

**Authors:** Carlos Henrique Valente Moreira, Ana Luiza Bierrenbach, Cesar Augusto Taconeli, Léa Campos de Oliveira-da Silva, Lewis F. Buss, Sheila M. Keating, Erika Regina Manuli, Noemia Barbosa Carvalho, Cristina Guastini, Sonia Bakkour Coco, José Ângelo Lauletta Lindoso, Lucas Augusto Moyses Franco, Fabio Ghilardi, Flavia Cristina da Silva Sales, Paul Contestable, Clara Di Germanio, Michael P. Busch, Ester Cerdeira Sabino

**Affiliations:** ^1^ Institute of Infectology “Emilio Ribas”, São Paulo, São Paulo, Brazil; ^2^ Division of HIV, Infectious Diseases, & Global Medicine- Zuckerberg San Francisco General Hospital, University of California, San Francisco, San Francisco, CA, United States; ^3^ Hospital Sírio-Libanês, Vital Strategies, São Paulo, São Paulo, Brazil; ^4^ Departamento de Estatística, The Federal University of Paraná, Curitiba, PR, Brazil; ^5^ Laboratório de Parasitologia Médica (LIM-46), Hospital das Clínicas da Faculdade de Medicina da Universidade de São Paulo (FMUSP), São Paulo, Brazil; ^6^ Centre for Academic Primary Care, University of Bristol, Bristol, United Kingdom; ^7^ GigaGen - A Grifols Company, San Carlos, CA, United States; ^8^ Departamento de Moléstias Infecciosas e Parasitárias, Hospital das Clínicas da Faculdade de Medicina da Universidade de São Paulo (FMUSP), São Paulo, Brazil; ^9^ Hospital das Clinicas de São Paulo, São Paulo, Brazil; ^10^ Vitalant Research Institute, San Francisco, CA, United States; ^11^ Grifols Diagnostic Solutions, Emeryville, CA, United States; ^12^ Ortho-Clinical Diagnostics, Inc, Raritan, NJ, United States; ^13^ Department of Laboratory Medicine, University of California, San Francisco, San Francisco, CA, United States

**Keywords:** Chagas, *Trypanossoma cruzi*, benznidazole, serology, PCR, biomarker

## Abstract

**Background:**

Evaluating the effectiveness of Chagas disease treatment poses challenges due to the lack of biomarkers for disease progression and therapeutic response. In this study, we aimed to assess the clearance of Trypanosoma cruzi (*T. cruzi)* parasites in a group of benznidazole (BNZ)-treated chronic Chagas disease patients using high-sensitivity quantitative PCR (qPCR) and track *T. cruzi* antibody levels through a semiquantitative chemiluminescent assay.

**Methods:**

A total of 102 *T. cruzi* seropositive patients with previous PCR-positive results were enrolled in the study. We collected samples 30 days before treatment (T-30d), on the day before initiating BNZ treatment (T0d), and at follow-up visits 60 days (T60d), 6 months (T6M), 12 months (T12M), and 36 months (T36M) after treatment initiation. Treatment efficacy was assessed by testing of serial samples using a target-capture qPCR assay specific to satellite *T. cruzi* DNA and the ORTHO *T. cruzi* ELISA Test System for antibody quantitation.

**Results:**

Of the enrolled individuals, 87 completed at least 50% of the treatment course, and 86 had PCR results at follow-up visits T6M, T12M, and T36M. PCR results exhibited fluctuations before and after treatment, but levels were significantly lower post-treatment. Only 15 cases consistently tested PCR-negative across all post-treatment visits. Notably, nearly all participants demonstrated a declining antibody trajectory, with patients who tested PCR-negative at T36M exhibiting an earlier and more pronounced decline compared to PCR-positive cases at the same visit.

**Conclusion:**

Our study suggests that serial PCR results pose challenges in interpretation. In contrast, serial antibody levels may serve as an ancillary, or even a more reliable indicator of parasite decline following BNZ treatment. Monitoring antibody levels can provide valuable insights into the efficacy of treatment and the persistence of parasites in Chagas disease patients.

## Introduction

More than 100 years after its discovery (Chagas, 1909), Chagas disease remains a major public health problem in South and Central America. Despite effective vector control, there are still hotspots of endemicity in countries such as Brazil, where one million people are estimated to be living with *Trypanosoma cruzi* (*T. cruzi*) infection (Brazilian Ministry of Health, 2022). Furthermore, due to human migration, around 400,000 people infected with *T. cruzi* live in non-endemic countries (Pinazo et al., 2011; Requena-Méndez et al., 2015). For most patients, *T. cruzi* infection is generally lifelong, and end-organ damage, such as progression to cardiac damage and other clinically significant sequelae, may occur over decades (Sabino et al., 2013). Therefore, millions of infected individuals could benefit from parasiticidal treatment to decrease this evolving process (Cardoso et al., 2018; Hasslocher-Moreno et al., 2021).

Benznidazole (BNZ) is the primary treatment available for Chagas disease worldwide. The evidence base supporting treatment with BNZ comes primarily from retrospective observational studies that have documented a reduced rate of progression to cardiomyopathy and lower mortality in BNZ-treated patients (Cardoso et al., 2018; Hasslocher-Moreno et al., 2021).

Surrogate markers of therapeutic success are urgently needed to allow more reasonable evaluation of existing treatments and routine clinical follow-up of BNZ-treated patients and support novel drug discovery and drug repurposing (Molina et al., 2014; Torrico et al., 2018; Pandey et al., 2022; Torrico et al., 2022). As evidenced by persistently negative polymerase chain reaction (PCR) test results, long-term sterilizing parasite clearance has previously been suggested as a surrogate for treatment success (Sulleiro et al., 2020). However, parasitemia is low-level, near the detection limit in untreated and chronically infected individuals, and may fluctuate in the absence of treatment (Morillo et al., 2015; De Salazar et al., 2022).

Consequently, persistently or intermittently negative results may not indicate parasite clearance after treatment. The presence of chronic parasitemia is linked to levels of anti-*T cruzi* antibodies, which are associated with the occurrence of abnormal electrocardiogram (ECG) results, positive results in quantitative polymerase chain reaction (qPCR) tests, as well as an increased risk of developing cardiomyopathy and long-term mortality. These findings highlight the potential value of measuring antibody quantities as an indicator of persistent parasite presence (Buss et al., 2020).

We present the three-year follow-up results of a prospective cohort of BNZ-treated chronic Chagas disease patients. We aimed to characterize the parasite clearance rate using a high-sensitivity quantitative qPCR assay and describe the trajectory of anti-*T. cruzi* antibody levels throughout early treatment and long-term follow-up relative to parasite persistence by PCR as a marker of therapeutic response.

## Methods

### Study design

**Figure 1:**
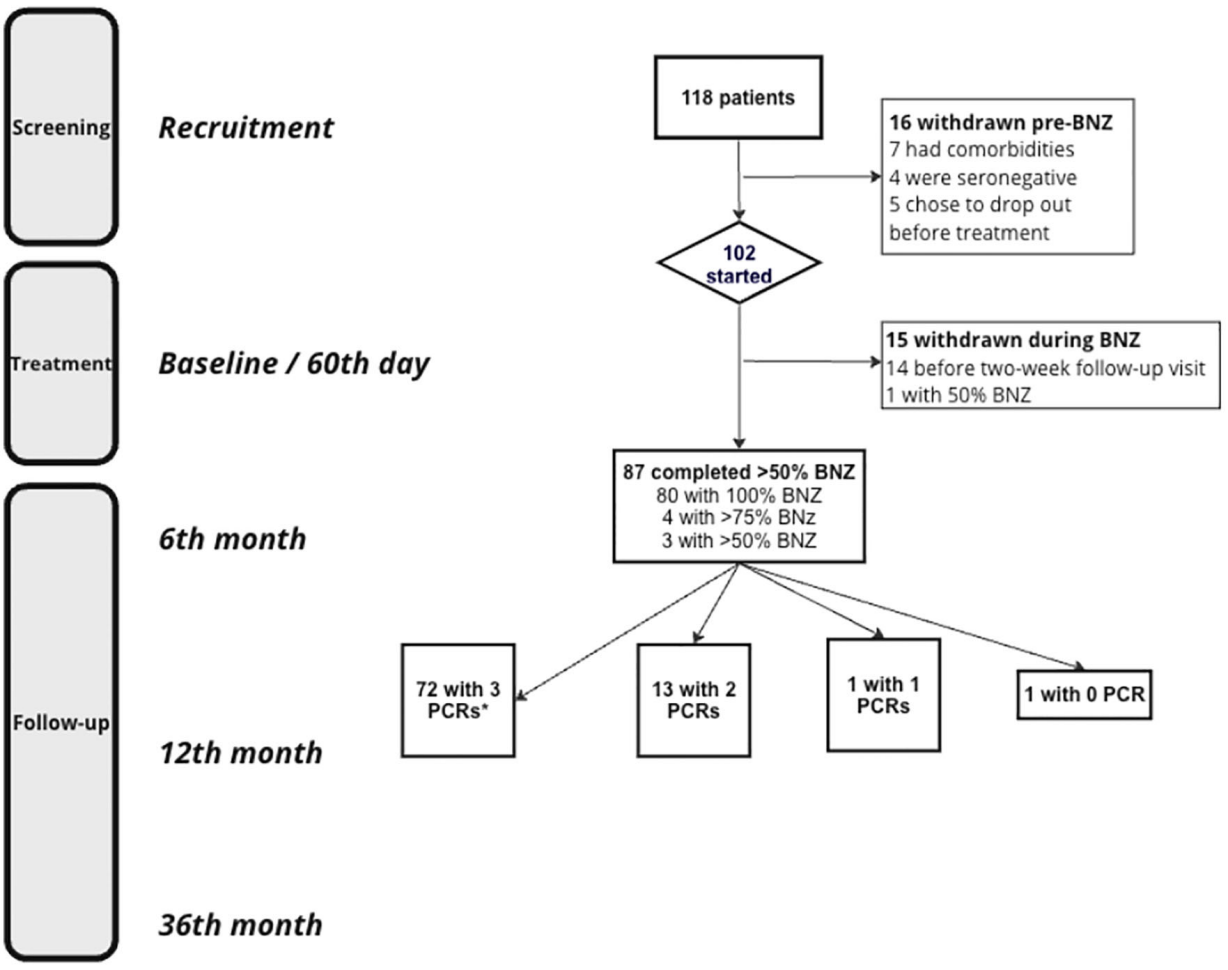
Study flowchart. *All follow-up PCRs are depicting those drawn after BZN treatment. BNZ, Benznidazole.

This is a prospective study, conducted from 2013 to 2015, of a cohort of chronic Chagas patients treated in outpatient services at two specialized centers in São Paulo, Brazil: Instituto de Infectologia “Emílio Ribas” and Hospital das Clínicas da Faculdade de Medicina da Universidade de São Paulo. Patients were enrolled before and followed up for three years after BNZ treatment. All individuals were PCR-positive before the treatment (**Figure 1**), and most were followed for three years following treatment with longitudinal clinical assessments and characterization of persistence and levels of *T. cruzi* parasitemia and antibodies.

### Study participants

We enrolled 102 individuals aged between 18 and 65 diagnosed with chronic Chagas disease and previously tested PCR-positive for *T. cruzi*. At different stages of the disease, these patients, ranging from indeterminate to mild cardiac or digestive involvement, self-reported and had their physicians confirm any comorbidities. They were then treated with BNZ at a standard dose of 5 mg/kg/day orally, up to a maximum of 300mg/day, split into two or three daily doses over 60 days (Dias et al., 2016).

### Data collection

We collected medical data and blood samples at multiple intervals. The first round, 30 days prior to the BNZ treatment (T-30d), included a detailed medical history, physical examination, and blood tests for *T. cruzi* qPCR and antibody quantitation. Another round of data collection occurred on the day before treatment initiation (T0d). We then gathered follow-up data at 60 days (T60d), six months (T6M), twelve months (T12M), and thirty-six months (T36M) post-treatment initiation, using the same data collection procedures.

### Blood processing

At the enrollment interview and follow-up visits, 12mL of blood anticoagulated with ethylenediaminetetraacetic acid (EDTA) was collected and immediately mixed with an equal volume of 6M guanidine/HCl-0.2M EDTA solution for PCR. These samples were boiled, aliquoted, and frozen at −20°C in Brazil. Serum separator tubes (SST) were also collected at each visit and centrifuged at 3000G for 15 min, and serum aliquots were frozen at -20°C. Aliquots of guanidine-lysed blood and serum were shipped to the US Central Laboratory (VRI; previously BSRI) for qPCR and serology, respectively.

### qPCR procedure

The qPCR assay used in this study was developed based on a PCR method that targets satellite *T. cruzi* DNA (DNAsat) (Sabino et al., 2015). Four replicate qPCR assays were performed from each of two DNA-extracted aliquots in eight replicates. For each assay, 500uL of guanidine-lysed blood was processed for DNA extraction using a target-capture (TC) step that employed magnetic beads coated with a *T.cruzi*-specific 20-mer capture oligonucleotides (Sabino et al., 2015). A qPCR cycle threshold (CT) of 45 was used as the cut-off. For qualitative interpretation, results were considered positive if at least two of four replicates were positive. Quantifying parasitemia was based on a standard curve derived from cultured parasites spiked into whole blood samples before lysis, with a quantitation range of 0.1 to 10,000 parasites/mL (Sabino et al., 2015).

### Serology procedure

The serological test (ORTHO *T. cruzi* VITROS ELISA Test System) is based on a whole parasite lysate antigen ELISA (Buss et al., 2020). The test utilizes microwells coated with T*. cruzi* antigens as the solid phase with chemiluminescent detection. Results are reported as signal-to-cutoff (S/CO) ratios representing the relative amount of anti-*T. cruzi* antibody present in test samples. Testing was performed according to the manufacturer’s instructions (Buss et al., 2020).

### Data analysis

Data from each patient were uploaded into REDCap (Research Electronic Data) (Harris et al., 2009; Harris et al., 2019), and results were then de-identified from specific patient-sensitive information.

We assessed changes in parasite loads measured through qPCR and serology levels by comparing the differences between the levels at multiple time points (T0d, T60d, T6M, T12M, and T36M) and the levels at T-30d. Our defined outcomes were as follows: “Negative” indicated a negative PCR result at T6M, T12M, and T36M; “Positive” indicated a positive result on the T36M visit, regardless of the earlier results; and “Inconclusive” referred to patterns that were negative on the T36M visit but had at least one PCR positive result at T6M or T12M visits. If the T6M or T12M PCR result was missing, we classified them as “Inconclusive”.

To analyze the data, we conducted a multinomial logistic regression with the Negative group as the reference, considering the age at study initiation and sex. We presented these outcomes graphically using a serial boxplot to show the trend across visits regarding the antibody levels between these groups.

To predict the likelihood of experiencing a “Non-Positive PCR outcome,” which includes the Negative and Inconclusive groups, we conducted a logistic regression with “Positive” as the reference category. The models were adjusted for the same variables as in the multinomial analysis. We presented the associations between predictor variables and the outcomes using odds ratios (OR) with 95% confidence intervals (CI 95%).

For qPCR and serology levels, we employed paired Wilcoxon rank-sum tests, paired t-tests, and Chi-squared tests with Bonferroni adjustment *post-hoc* for comparison across visits and the T0d visit. We used the Kruskal-Wallis test to analyze the differences in antibody levels across visit time points and outcomes for *T. cruzi*-infected patients treated with benznidazole. Continuous variables were reported as medians and interquartile ranges or mean and standard deviations based on their distribution, while categorical variables were reported as frequencies and percentages.

Our conclusions are based on a two-tailed hypothesis test with an alpha error probability of 0.05. We performed the statistical analysis and generated graphics using R Studio software (version 4.2) (RStudio, 2022).

### Ethics statement

This study protocol was approved by the local ethics committee of the Hospital of Clinics, University of São Paulo, and Institute of Infectology “Emilio Ribas” (CAAE00580612.8.0000.0065). All patients provided written informed consent at the time of recruitment.

## Results

From the total of 102 patients treated for Chagas disease, a subset of 87 patients who completed at least 50% of the BNZ treatments were considered for the initial analysis. A more detailed breakdown of this subset was further assessed for informative data: out of these 87 patients, 86 had both qPCR and antibody results available from their follow-up visits at six months (T6M), twelve months (T12M), and thirty-six months (T36M). These 86 patients were included in the final data analysis (**Figure 1**). The cohort was predominantly male (62%) with a median age of 53 years (interquartile range of 47 to 59 years). Among these patients, 34 (39.1%) were diagnosed with a mild cardiac form of Chagas disease (**Table 1**).

**Table 1:** Clinical characteristics of the subjects that completed at least 50% of the BZN course.

Variables	N = 87 (%)
Age (years) 35 – 44 45 – 54 55 – 64	17 (19.5) 32 (36.8) 38 (43.6)
Sex: Male	54 (62.1)
Race/skin color Black More than one race White	13 (14.9) 51 (58.6) 23 (26.4)
Literate: yes	77 (88.5)
Comorbidities Hypertension Diabetes Chronic kidney disease	30 (34.5) 11 (12.6) 1 (1.1)
Chagas disease form* Cardiac Indeterminate Digestive	34 (39.1) 28 (32.2) 29 (33.3)

*4 cases had both Cardiac and Digestive form.

**Figure 2:**
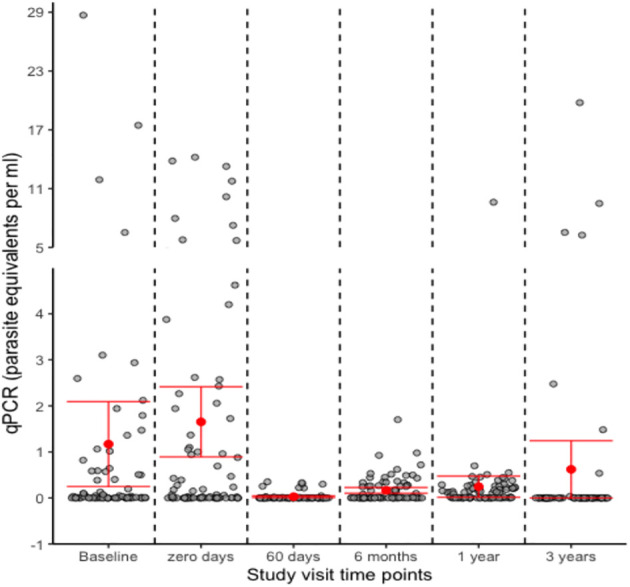
Dotplot representing individual Polymerase Chain Reaction (qPCR) measurements for 85 subjects treated with benznidazole, with at least three follow-up PCR measurements. The red dots represent the mean values, while the red bars indicate the 95% confidence interval around the mean. Note the different scales in the two segments of the y-axis.

qPCR levels fluctuated during the two pre-treatment visits, with approximately 30% of the patients having parasitemia levels higher than one parasite equivalent/mL and 45% having PCR-negative results at one of these two visits on post-study batch testing at the Central Lab. Following treatment, the qPCR levels continued to fluctuate, particularly at T6M and T12M, but were significantly lower than pre-treatment levels and the T60d visits, as shown in **Table 2**. Our study found that qPCR levels showed a statistically significant decrease compared to baseline (T0d) measurements, starting from T60d in patients with a non-positive outcome. This significant decrease persisted over time. On the other hand, patients with a positive outcome experienced a statistically significant drop in qPCR levels only at their final follow-up visit (T36M) compared to their T0d levels. Only 15 patients had negative PCR results on all their ≥T6M post-treatment follow-up visits. The proportion of patients with PCR positivity across the follow-up visits varied, with a significant difference from the initial visit (T0d) at the T60d but not the T6M and T12M visits, and only regaining significance at the last visit (T36M). The fluctuation of the PCR results made it difficult to determine which patients had achieved parasite suppression. Interestingly, on the T36M visit, 90% of the patients had negative PCR results (**Table 2**; **Figure 2**).

**Table 2:** PCR and serological results of Chagas patients completing at least 50% of the treatment course with benznidazole.

Variables	Study time point					
	Baseline	Day 0	Day 60	6 months	1 year	3 years
Number of patients with PCR results	84	84	85	85	83	75
PCR-positive, n (%)*	44 (52)	45 (54)	19 (22)	37(44)	46(55)	8(11)
PCR positivity compared to day zero visit (p-value)**	1.0	Reference	<0.001	1.0	1.0	<0.001
Median qPCR and IQR (parasite eq./ml)	0.016 (0 - 0.51)	0.028 (0 -1.29)	0.0 (0-0.004)	0.009 (0-0.265)	0.105 (0-0.214)	0.0 (0)
qPCR compared to day zero*** (p-value)	0.203	Reference	<0.001	0.002	0.036	0.002
PCR-parasite eq/mL <0.01 to 0.09 to 0.99 ≥1.0	39 (45) 17 (20) 14 (17) 14 (17)	37 (46) 10 (12) 14 (17) 23 (27)	66 (78) 8 (9) 11 (13) 0(0)	44 (52) 8 (9) 32 (38) 1(1)	34(41) 6(7) 42(50) 1(1)	67(90) 1(1) 1(1) 6(8)
Serology S/N mean and SD	10.3 (3.3)	10.4 (3.3)	9.8 (3.1)	9.3 (3.0)	8.8 (3.1)	7.2 (2.6)
Serology compared to Baseline values**** (p-value)	NA	0.121	0.001	<0.001	<0.001	<0.001

****Paired T-Test; *** Paired samples Wilcoxon test; **Chi-squared test; *n=33 (38%) were PCR-positive in both, T-30d and T0d.

NA, Not Applicable.

**Figure 3:**
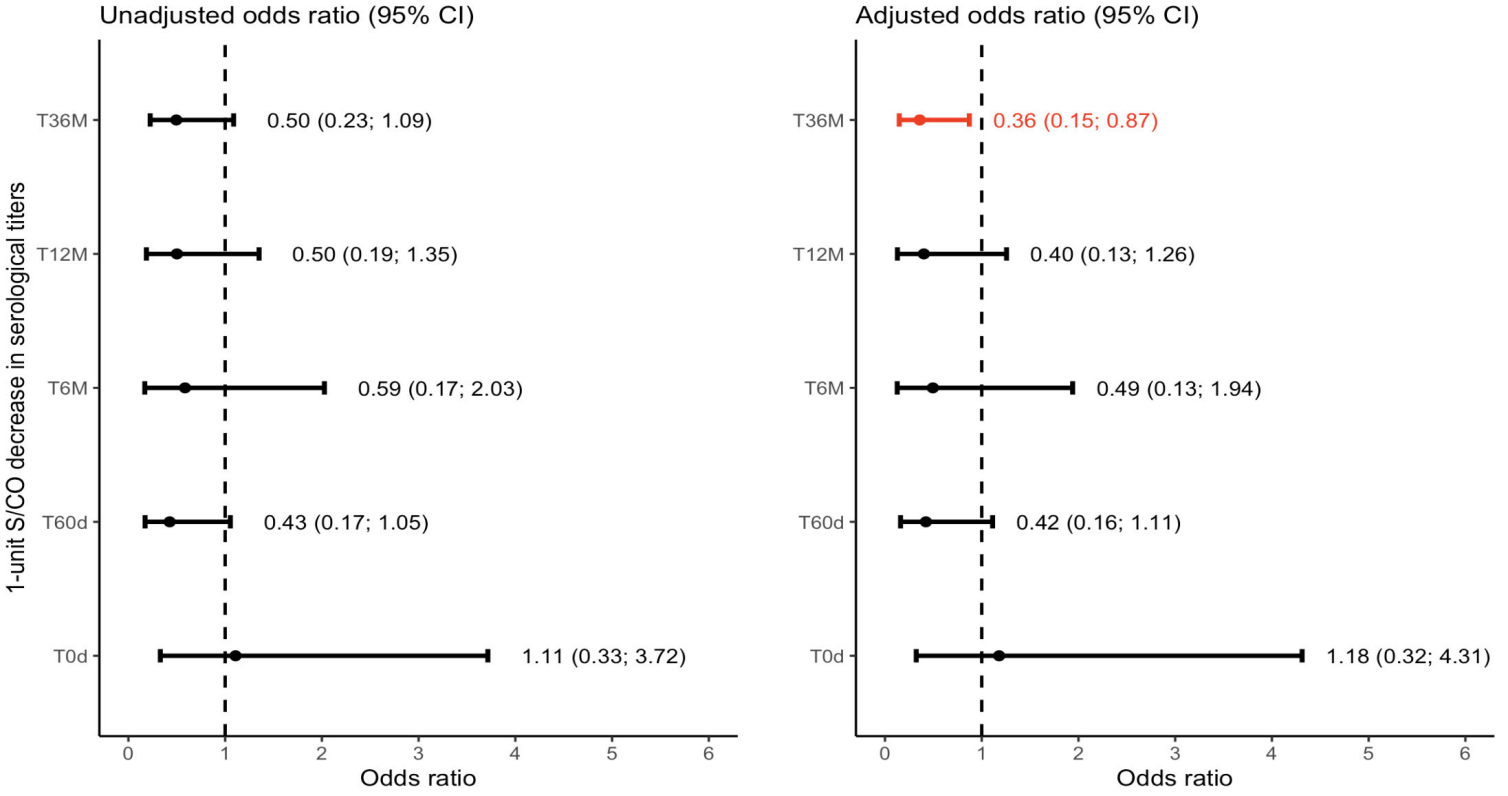
Forest plot depicting the Multinomial Logistic Odds Ratio for the positive outcome group with negative as the reference category. CI: Confidence Interval; S/CO: Signal-to-Cutoff.T0d: Time 0 day; on the day at benznidazole treatment initiation. T60d: Time 60 days; follow-up visit 60 days after initiating benznidazole treatment. T6M: Time 6 Months; follow-up visit 6 months after initiating benznidazole treatment; T12M: Time 12 Months; follow-up visit 12 months after initiating benznidazole treatment. T36M: Time 36 Months; follow-up visit 36 months after initiating benznidazole treatment.

Our multinomial logistic regression analysis revealed that the difference in antibody (Ab) levels after treatment, compared to baseline levels, could predict the odds of not belonging to the Positive outcome category. Specifically, at the T36M visit, a 1-unit decrease in Ab values was significantly associated with a reduced likelihood of being classified as part of the Positive outcome group relative to the Negative reference category. The odds decreased by a factor of 0.36, indicating that each unit decrease in Ab levels was associated with a 64% decrease in the likelihood of belonging to the Positive outcome category (**Figure 3**). Similar trends were observed at the T12M and T6M visits. At the T12M visit, a 1-unit decrease in Ab levels, compared to the T-30d visit, was associated with a 60% reduction in the odds of being classified as part of the Positive outcome group, compared to the Negative group. Similarly, at the T6M visit, a 1-unit decrease in Ab levels resulted in approximately a 51% decrease in the odds of being classified as part of the Positive outcome group. Interestingly, the drop in S/CO between T-30d and T60d is predictive of the outcome at the T36M visit. However, the CI95% was too large to rule out the possibility of no effect.

**Figure 4:**
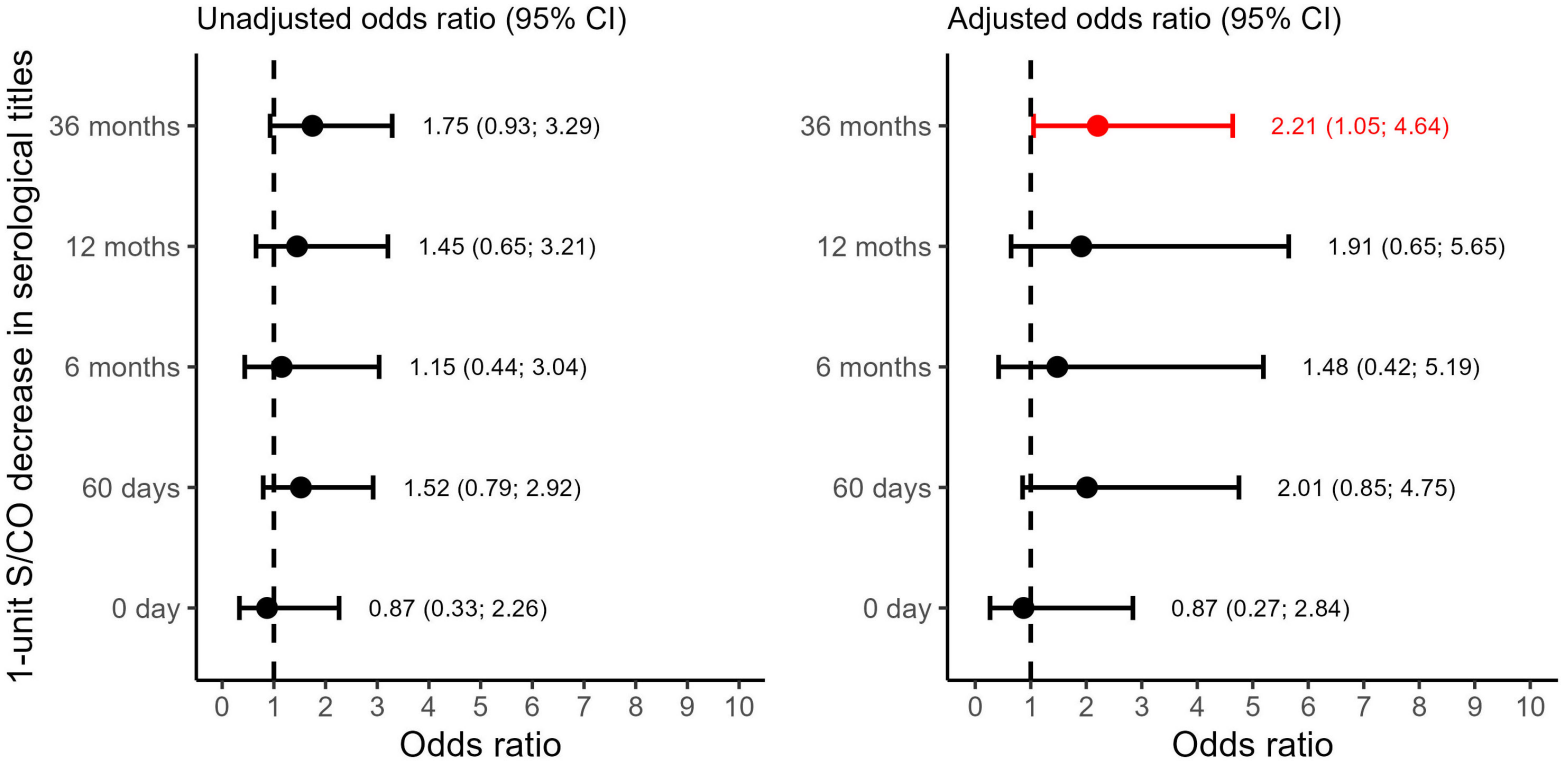
Forest plot depicting the Binomial Logistic Odds Ratio for the non-positive result (negative or inconclusive) outcome group with positive as the reference category. CI: Confidence Interval. S/CO: Signal-to-Cutoff. T0d: Time 0 day; on the day at benznidazole treatment initiation. T60d: Time 60 days; follow-up visit 60 days after initiating benznidazole treatment. T6M: Time 6 Months; follow-up visit 6 months after initiating benznidazole treatment; T12M: Time 12 Months; follow-up visit 12 months after initiating benznidazole treatment. T36M: Time 36 Months; follow-up visit 36 months after initiating benznidazole treatment.

Our study also investigated the association between qPCR results, antibody levels, and the probability of attaining a Non-Positive outcome comprising Negative or Inconclusive results. The results revealed a consistent trend of higher success rates in parasite suppression over time, with the likelihood more than doubling at the T36M visit compared to earlier time points among those presenting the reference category as “Positive” (**Figure 4**).

**Figure 5:**
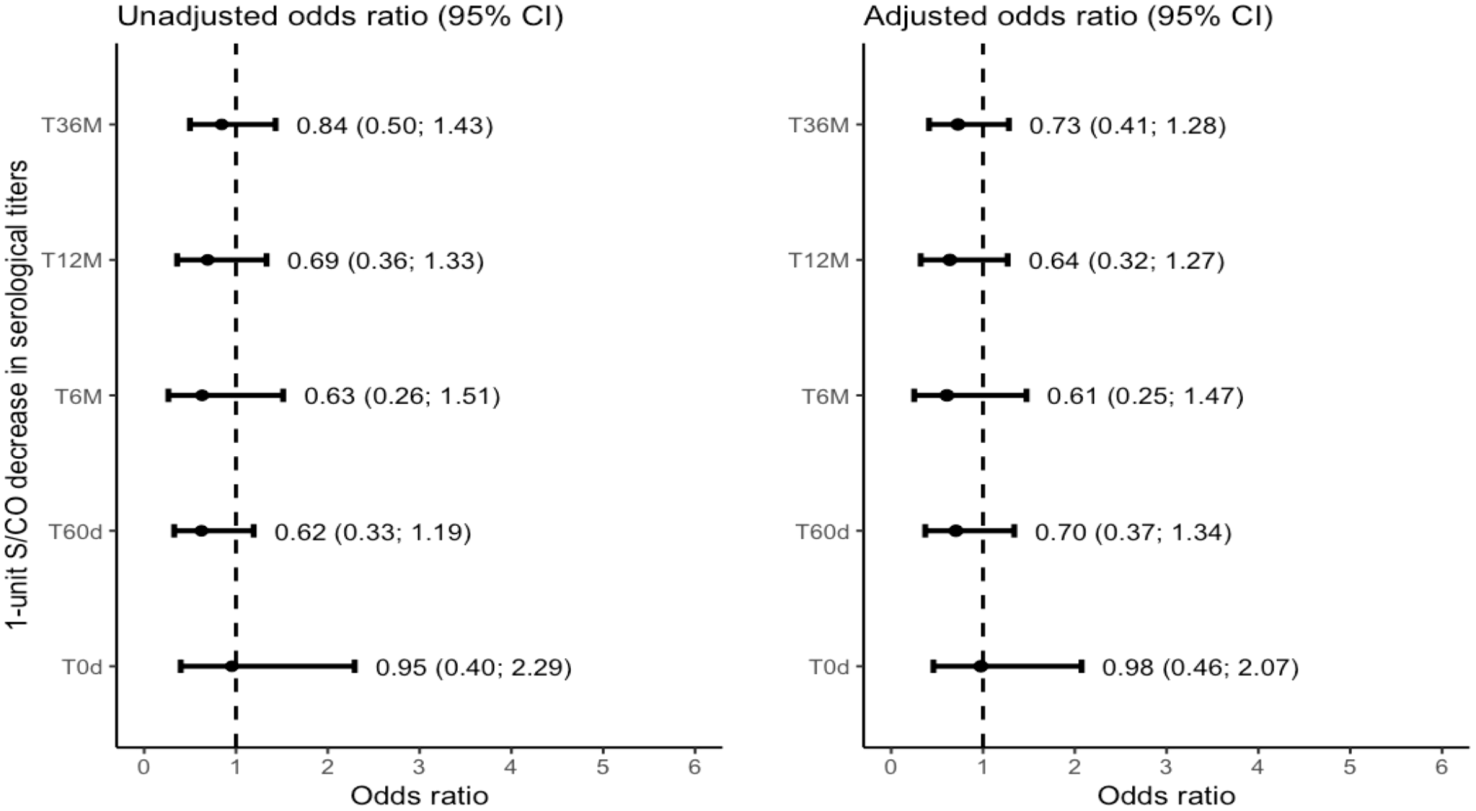
Forest plot depicting the Multinomial Logistic Odds Ratio for the inconclusive outcome group with negative as the reference category. CI: Confidence Interval. S/CO: Signal-to-Cutoff. T0d: Time 0 day; on the day at benznidazole treatment initiation. T60d: Time 60 days; follow-up visit 60 days after initiating benznidazole treatment. T6M: Time 6 Months; follow-up visit 6 months after initiating benznidazole treatment; T12M: Time 12 Months; follow-up visit 12 months after initiating benznidazole treatment. T36M: Time 36 Months; follow-up visit 36 months after initiating benznidazole treatment.

**Figure 6:**
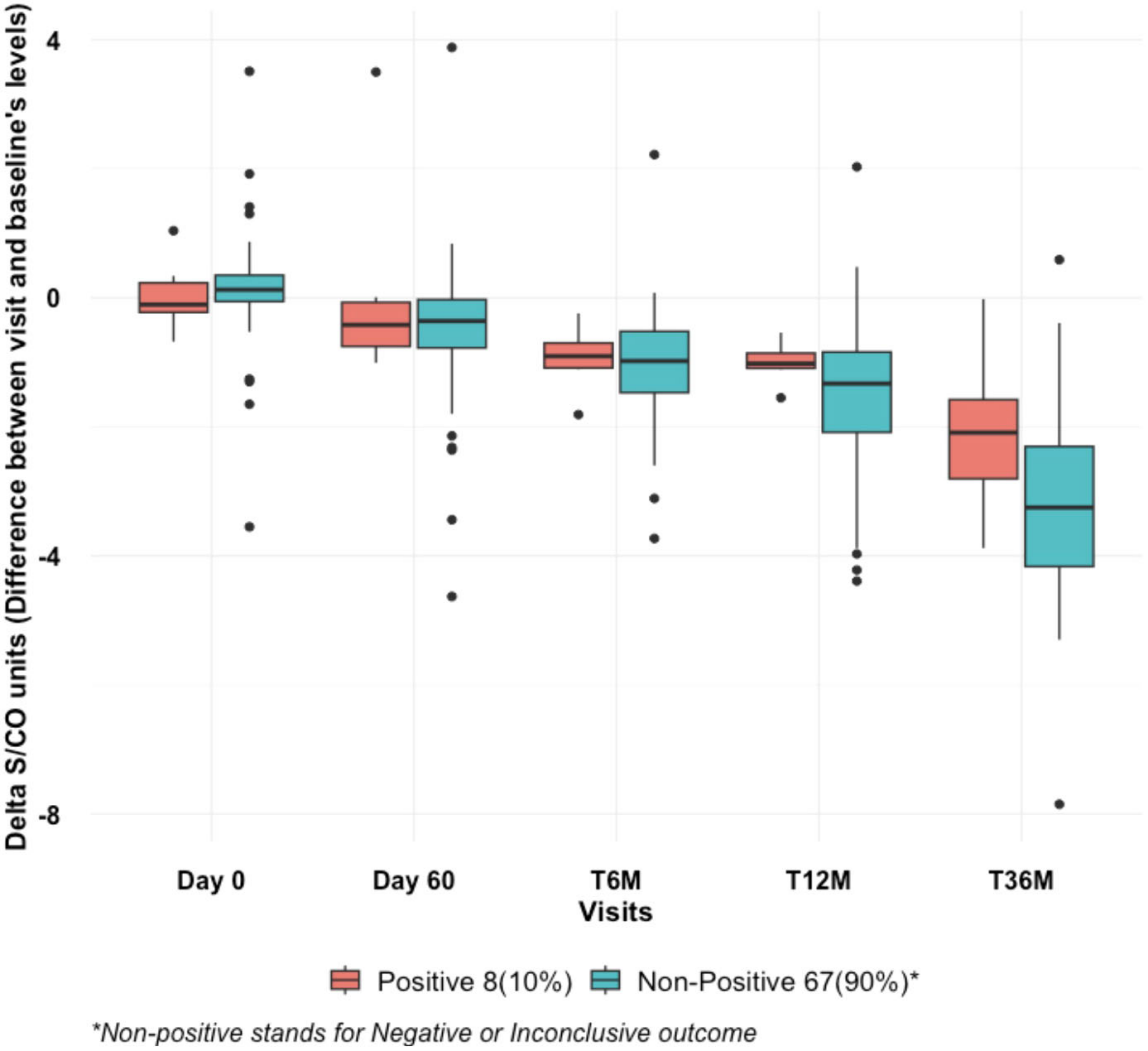
Serial boxplot over visits depicting Decaying Serological Values (S/CO): from Baseline across follow-up visits between defined outcomes. Positive vs. Non-Positive Outcomes (negative or inconclusive). S/CO: Signal-to-CutOff; T0d: Time 0 day; on the day at benznidazole treatment initiation. T60d: Time 60 days; follow-up visit 60 days after initiating benznidazole treatment. T6M: Time 6 Months; follow-up visit 6 months after initiating benznidazole treatment. T12M: Time 12 Months; follow-up visit 12 months after initiating benznidazole treatment. T36M: Time 36 Months; follow-up visit 36 months after initiating benznidazole treatment.

**Figure 7:**
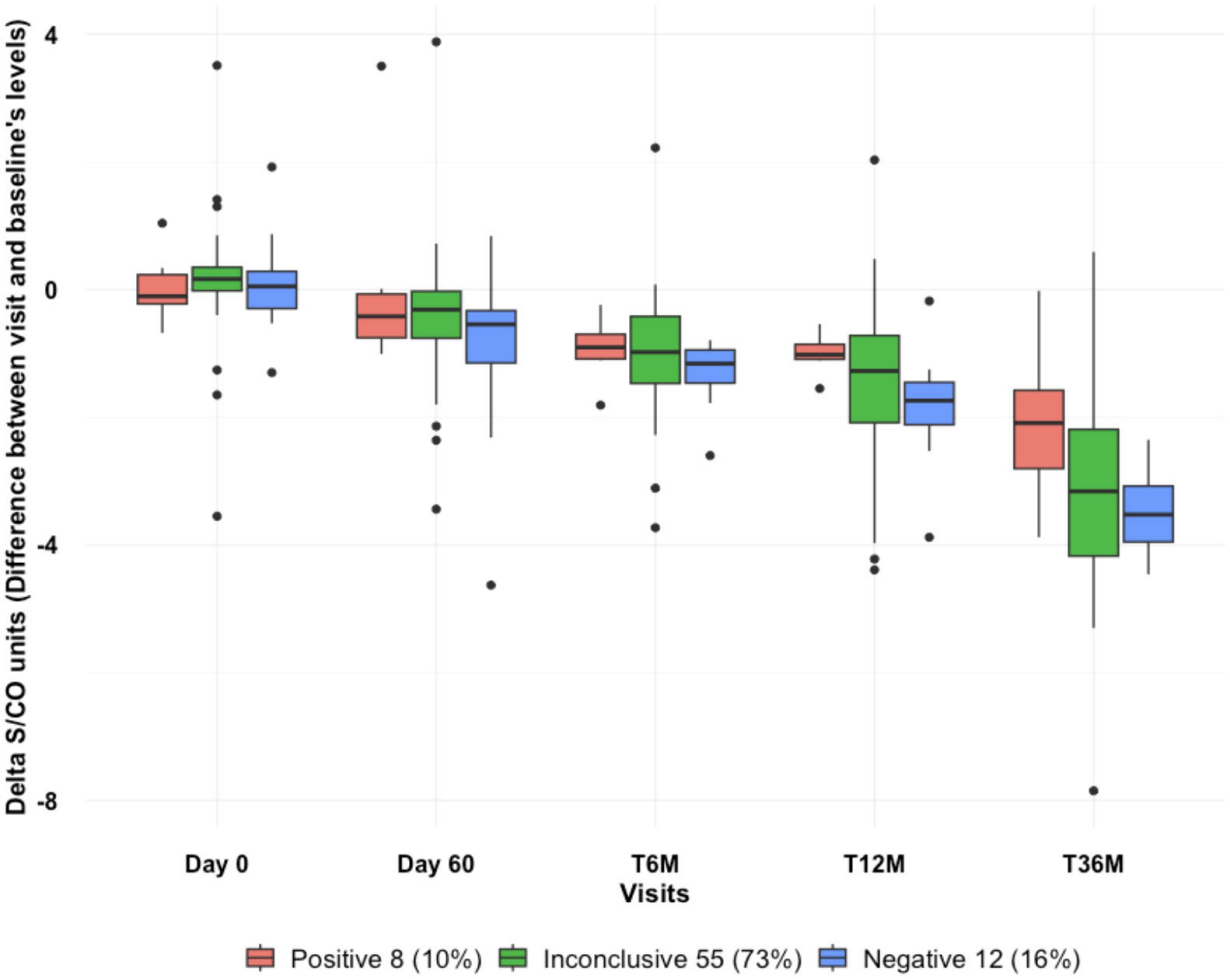
Serial boxplot over visits depicting Decaying Serological Values (S/CO): from Baseline across follow-up visits between defined outcomes: Combined PCR outcome (T6M, T12M, T36M), respectively: Negative (negative result in all visits); Positive (positive result at the T36M visit, regardless of the previous results); Inconclusive (negative result at the T36M visit, and at least one positive result at T6M or T12M).

**Figure 8:**
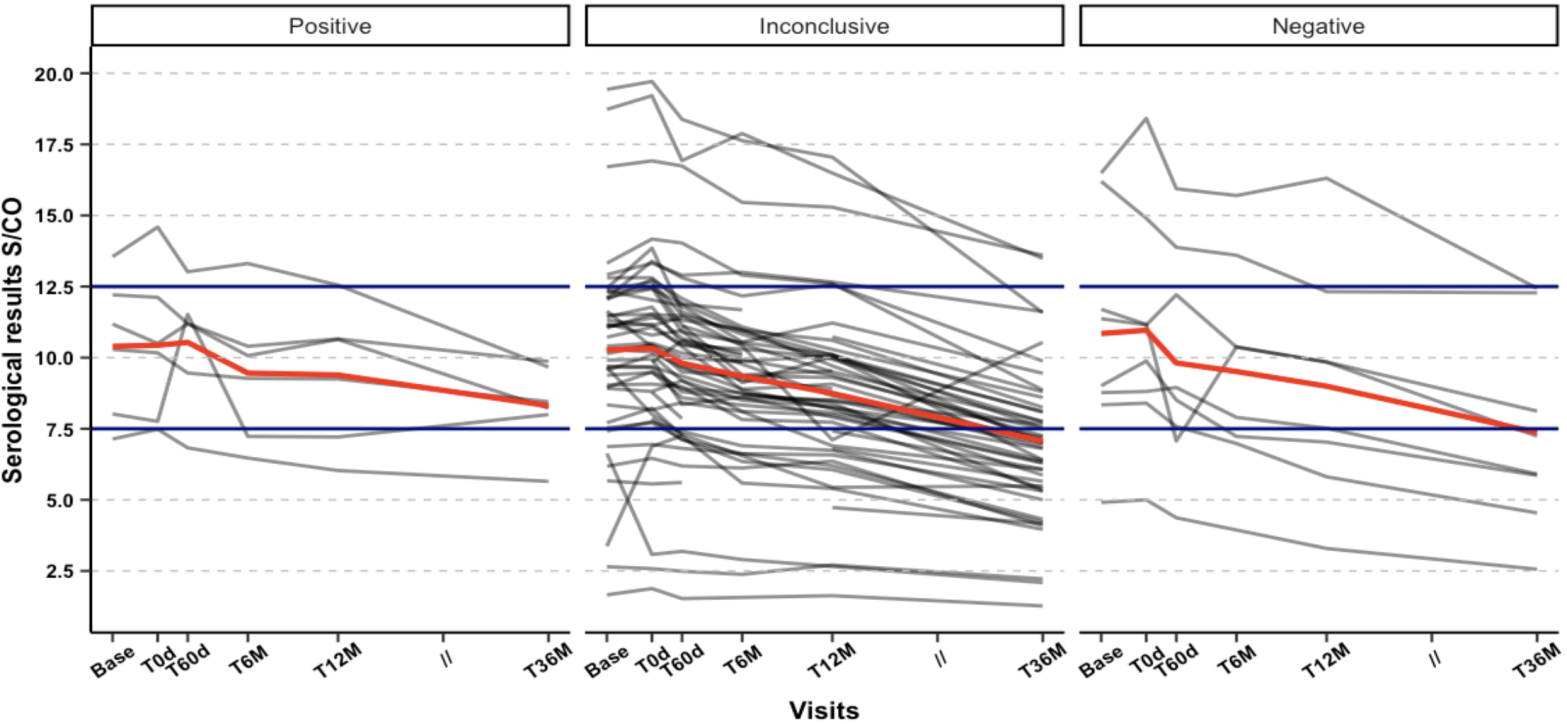
Individual Serological values (S/CO) trajectories from Baseline across follow-up visits between defined outcomes: Combined PCR outcome (T6M, T12M, T36M), respectively: Negative (negative result in all visits); Positive (positive result at the T36M visit, regardless of the previous results); Inconclusive (negative result at the T36M visit, and at least one positive result at T6M or T12M).

However, it is important to consider the wider confidence intervals observed in most of the observations, which make it challenging to rule out the possibility of no effect conclusively. These wider intervals can be attributed to the limited number of data points and observations across the Positive outcome group. Despite this limitation, we consistently observed a significant decline in antibody levels among patients who were classified within the Negative group. This trend was observable in both approaches for grouping the outcomes, even when we split the patients into a third group, the Inconclusive. This trend was noticeable as early as the T6M visit (**Figures 5**–**8**).

## Discussion

In our study, we tracked 87 patients infected with *T. cruzi*, all of whom were treated with BZN. The PCR results we obtained were challenging to interpret, primarily when used to determine complete parasite elimination. Most patients still showed detectable levels of PCR at least once post-treatment, even though these levels were significantly lower than before treatment. Interestingly, we observed a consistent and early decrease in antibody levels, especially in those patients classified as the ‘Negative’ group.

Our PCR methodology incorporated several steps to enhance sensitivity, including a larger whole blood volume for extraction of 500uL (2 x 250uL) extraction, target capture assay, and eight replicates per sample. This may explain why our study differs from other clinical trials that did detect higher BZN responses defined by earlier and sustained PCR results (Morillo et al., 2015; Morillo et al., 2017). Another plausible explanation is that the older age of our cohort could have contributed to the lower efficacy of BZN. Immunosenescence may play a role in unbalanced parasite clearance since it can interfere with developing protective humoral immunity in response to infectious pathogens in older people (Crooke et al., 2019). Also, we can speculate that the strains present in this population were more resistant to the drug. However, we can not corroborate this since our study did not aim to perform parasite typing or check BNZ resistance. However, interestingly, recent studies have challenged the parasite resistance to the BNZ concept and its association with unsuccessful parasitological outcomes by proposing that this resistance is relative, incomplete, and not associated with specific genetic parasite lineages or host genetics and that the unpredictable treatment outcomes may be due to a transiently dormant amastigote phase (Sánchez-Valdéz et al., 2018).

Interpreting the PCR results posed challenges, but after three years, an encouraging 90% of patients tested negative, indicating that some may have achieved parasite suppression. The proportion of positive results varied significantly between the initial visit (T0d) and subsequent visits, particularly at T60d, which coincided with the end of treatment and the anticipated peak of benznidazole’s trypanocidal effect. However, this significant difference was not consistently observed during the T6M and T12M visits, suggesting potential fluctuations in detectable parasitemia by PCR. Nevertheless, statistical significance was regained at the final follow-up visit (T36M), indicating likely parasite clearance in certain patients (refer to **Table 2**). While qPCR levels showed a decline across all treatment time points, we agree that these levels should not be regarded as a standalone marker for treatment effectiveness. This is because the qPCR levels did not differentiate between the treatment groups at individual time points. Notably, our study revealed that participants with a >1 S/CO drop at T12M were all negative on PCR at the three-year mark, indicating that an early decrease in antibody titers may be an early predictor of long-term parasite clearance and treatment success.

One limitation of our study is the lack of a non-treated control group, as it would have been ethically challenging to maintain such a group for an extended period. Studies have shown spontaneous antibody decline may occur in untreated patients, however, only in a small proportion of the patients and after a more extended time (Buss et al., 2020). Therefore, the observed antibody decline was likely due to BZN treatment rather than natural variation. Secondly, we didn’t include those patients who had taken less than 50% of the prescribed drug in our analysis, decreasing the study sample. However, we should have included them since recent studies have proposed a shorter BNZ trend compared with the standard treatment (Torrico et al., 2021) and a better adverse effects profile.

In conclusion, our study highlights the challenges of interpreting *T. cruzi* PCR results. It suggests that antibody decline detected by chemiluminescent assay may be a valuable marker for BZN therapeutic response. Further follow-up visits are necessary to fully understand the long-term response to BZN treatment considering the association of such parasitological outcomes and antibody dynamics.

## Data availability statement

The original contributions presented in the study are included in the article/**Supplementary Material**. Further inquiries can be directed to the corresponding author.

## Ethics statement

The studies involving human participants were reviewed and approved by Ethics committee of the Hospital of Clinics, University of São Paulo, and Institute of Infectology “Emilio Ribas” (CAAE00580612.8.0000.0065). The patients/participants provided their written informed consent to participate in this study.

## Author contributions

Conceptualization: ES, AB, CM, MB. Data Curation: CM, CT, LB, SK, CDG, PC. Formal Analysis: CM, CT, AB, LB, CT, SC. Funding Acquisition: ES, MB. Investigation: CM, FG, CG, NC, JL, LS, FS, EM, LF, FG. Methodology: LS, FS, EM, LB, CM, CT, CDG, FG, PC. Project Administration: ES, LS, MB, SK. Resources: ES, LS, MB, EM, PC. Software: CM, CT, LF, FG, LB. Validation: LS, LF, FG, AB. Visualization: CM, ES, AB, MB, LB. Writing – Original Draft Preparation: CM, LB. Writing – Review & Editing: all authors. All authors contributed to the article and approved the submitted version.
