# Supplementary figures and images for: Parasitemia and antibody response to benznidazole treatment in a cohort of patients with chronic Chagas disease
Source: Front Parasitol. 2023 Sep 5;2:1235925. doi: 10.3389/fpara.2023.1235925 (PMC11731785; doi:10.3389/fpara.2023.1235925)

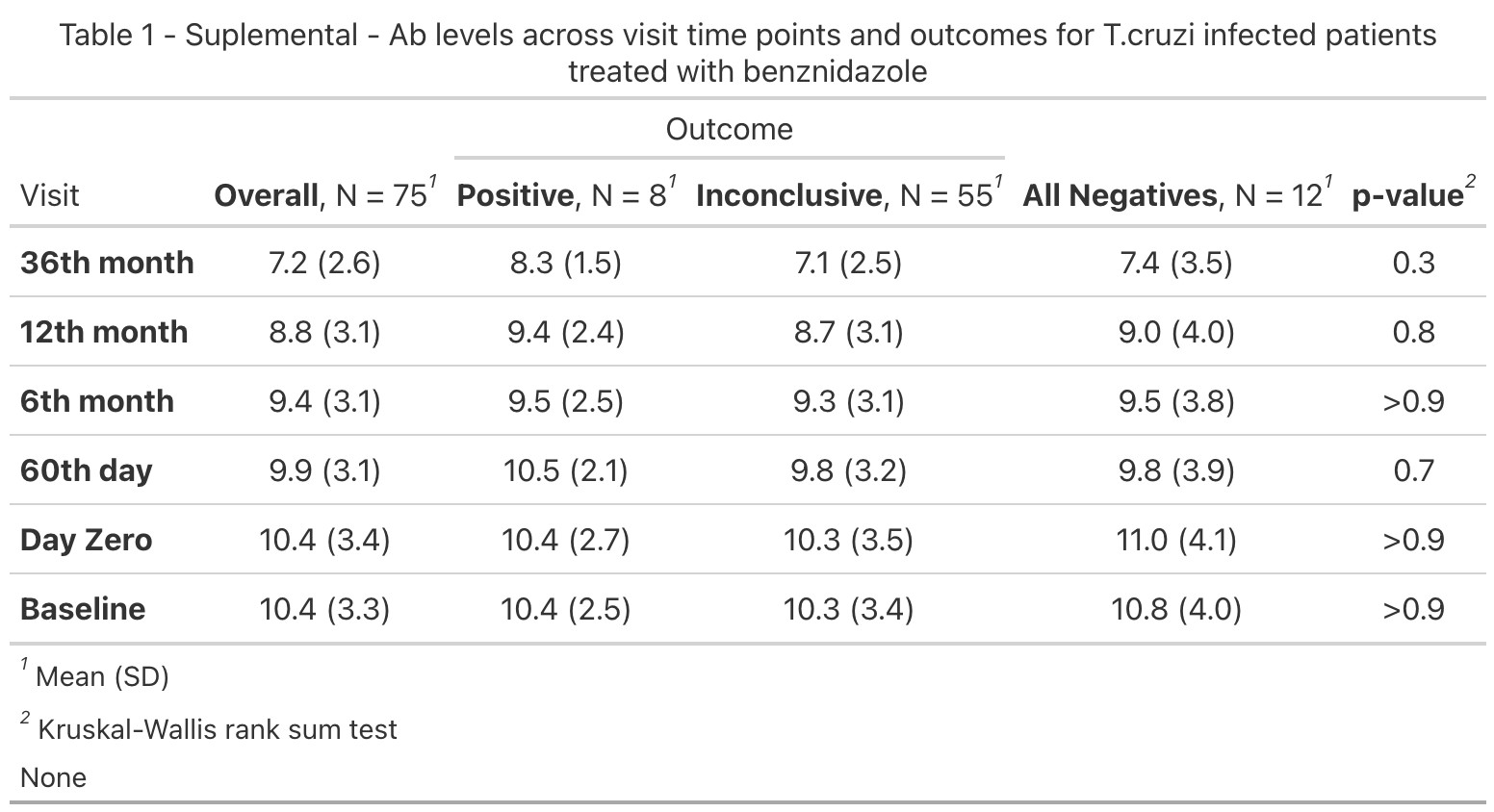

Supplement: Supplementary file 3 [file Table_1.docx]

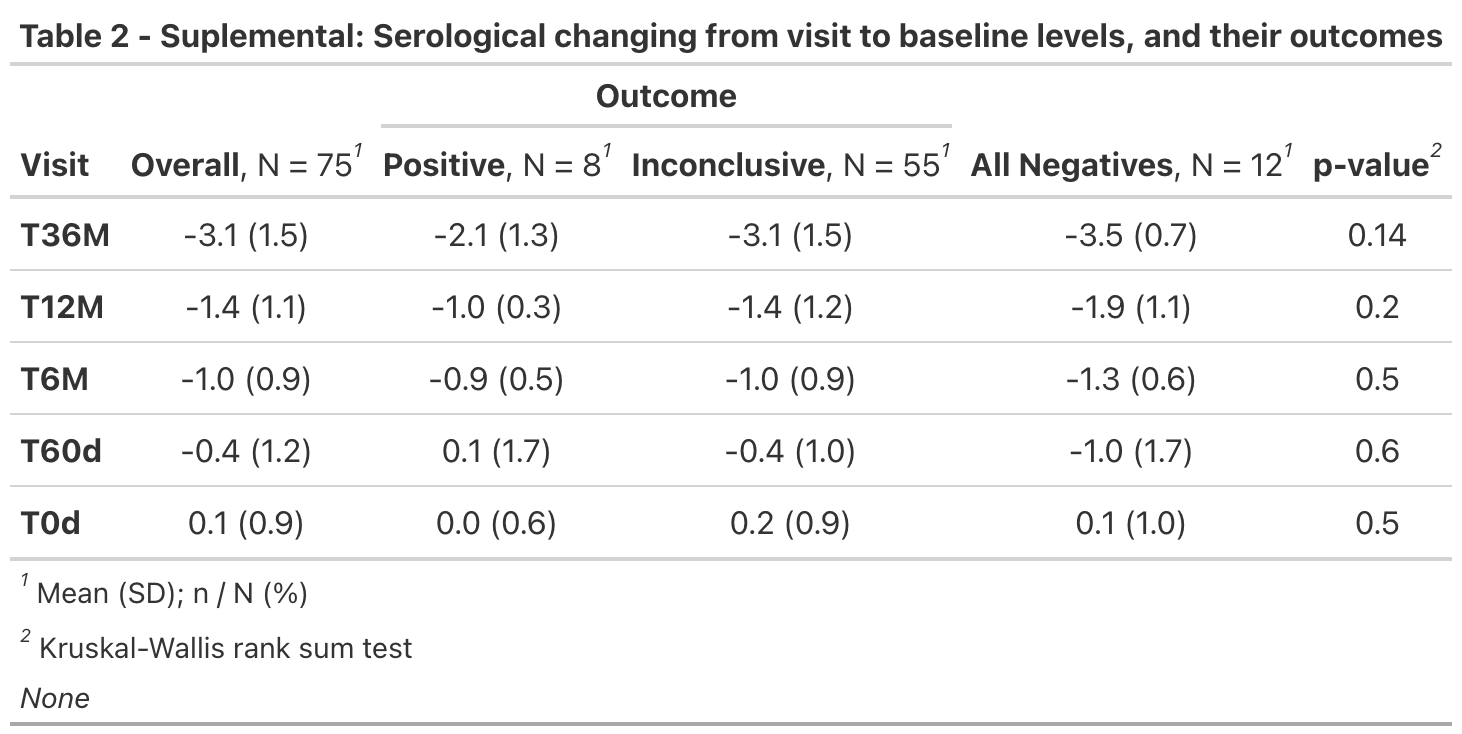

Supplement: Supplementary file 4 [file Table_2.docx]
